# In silico identification and validation of miRNA and their DIR specific targets in *Oryza sativa* Indica under abiotic stress

**DOI:** 10.1016/j.ncrna.2020.09.002

**Published:** 2020-09-18

**Authors:** Deepak Kumar Singh, Shourya Mehra, Sayan Chatterjee, Ram Singh Purty

**Affiliations:** University School of Biotechnology, Guru Gobind Singh Indraprastha University, Sec-16C, Dwarka, New Delhi, India

**Keywords:** Oryza sativa, Dirigent proteins, Abiotic stress, microRNA, Gene expression, Motif analysis, Molecular phylogeny

## Abstract

Several biotic (bacterial and viral pathogenesis) and abiotic stress factors like salt, drought, cold, and extreme temperatures significantly reduce crop productivity and grain quality throughout the world. MicroRNAs (miRNAs) are small (~22 nucleotides) non-coding endogenous RNA molecules which negatively regulate gene expression at the post-transcriptional level either by degrading the target protein-coding mRNA genes or suppressing translation in plants. Dirigent (DIR) gene protein plays a crucial role as they are involved to dictate the stereochemistry of a compound synthesized by other enzymes as well as in lignifications against biotic and abiotic stress. In plants, several miRNAs, as well as their targets, are known to regulate stress response but systematic identification of the same is limited. The present work has been designed for *in silico* identification of miRNAs against a total of sixty-one DIR genes in *Oryza sativa* Indica followed by target prediction of identified miRNAs through the computational approach and thereafter validation of potential miRNAs in rice genotypes. We systematically identified 3 miRNA and their respective DIR specific target gene in *Oryza sativa* Indica. The expression of these three miRNAs and their respective DIR specific targets were validated in rice seedlings subjected to five different abiotic stress conditions (heavy metal, high temperature, low temperature, salinity and drought) by quantitative Real-Time PCR (qRT-PCR). Expression analysis indicated that miRNA under stress conditions regulates the gene expression of the DIR gene in rice. To the best of our knowledge this is this is the first report in any organism showing the expression of ath-miRf10317-akr, and osamiRf10761-akr miRNAs in response to various abiotic stresses.

## Highlights

• Total 61 DIR proteins were identified & classified into 6 groups based on phylogeny analysis in Oryza sativa Indica.
• Three miRNAs ath-miRf10317-akr, cre-miR910 and osa-miRf10761-akr were identified via computational approach.
• These 3 miRNAs in response to abiotic stresses showed inverse expression pattern in the respective target genes.
• This is the first report on expression of ath-miRf10317-akr, and osa-miRf10761-akr miRNAs in response to abiotic stresses.

## 1 Introduction

Rice (*Oryza sativa* L.) is one of the major cereal grain crops of the developing countries and the staple food of around 78% of the world's population. Productivity of rice is greatly affected due to various abiotic stresses which includes salinity, drought, heavy metal and extreme temperature condition. In response to various abiotic and biotic stresses, plants synthesize several proteins including the dirigent proteins (DIRs) [1].

Dirigent proteins are extracellular glycoproteins with high β-strand content and have been found in all vascular plants, including lichens, ferns, gymnosperms and angiosperms [[2], [3], [4]]. DIRs involved in secondary metabolism, lignan and lignin biosynthesis [5,6]. DIRs were found to mediate regio- and stereoselectivity of bimolecular phenoxy radical coupling during lignin biosynthesis [[7], [8], [9]]. Exposure to abiotic stress lead to the modulation of lignifications levels which is an implication of DIRs and peroxidases [1]. The expression of the most responsive DIRs was found to be correlated with increased lignifications when studied for cold stress [10,11]. In soybean roots, the peroxidase activity and wall lignifications get enhanced under stress due to manganese (Mn) toxicity [12]. Application of drought, salt, and oxidative stress resulted in the exhibition of stem specific expression by a *Saccharum* spp. DIR gene (ScDIR) [13]. In *Medicago sativa*, transcriptional up-regulation of one DIR gene was observed under heat stress whereas transcriptional down-regulation of two peroxidases and another DIR was observed under cold stress [14].

In plants, many studies have revealed that microRNAs (miRNAs) play a vital post-transcriptional regulatory role in gene expression by target mRNA cleavage or translational inhibition [15]. In plants, mature miRNAs are generated from the long stem-loop precursor (pre-miRNAs) by a DICER-like RNA endonuclease and then the RNA-Induced Silencing Complex (RISC) guided by ARGONAUTE 1 (AGO1) protein directs the miRNA to the complementary target mRNA sequence [[15], [16], [17], [18]]. Plant miRNAs are reported to possess important functions in several metabolic and biological pathways such as tissue development and differentiation, biotic and abiotic stress responses, phytohormones signaling, and secondary metabolite production [19,20]. Nonetheless, the evolutionary highly conserved nature of an extensive number of miRNAs simplified the process of characterization of novel miRNA orthologs in new plant species through homologs identification [21]. Several abiotic stress-sensitive miRNAs have been reported over a period of time in various studies, e.g. In *Arabidopsis thaliana,* miRNA398 is involved in oxidative stress tolerance [22], and gene expression of 21 miRNAs are up-regulated in response to UV-B exposure [23].

Knowing the importance of miRNA and their roles in gene regulation, in the present investigation, an experiment has been designed for *in silico* identification of miRNAs and their potential DIR targets in rice through computational approach and validation of putative miRNAs using quantitative real-time PCR (qRT-PCR) under different abiotic stress condition.

## 2 Materials and methods

### 2.1 Identification and domain analysis of DIR family genes in rice

We identified candidate DIR family genes by using their respective Pfam ID i.e., “PF03018” against the rice genome database in the Ensembl Plant (http://plants.ensembl.org/Oryza_indica/Info/Index). The amino acids, as well as the cDNA sequence of all the selected DIR proteins, were then retrieved from the Ensembl Plant database for further analysis. The amino acid sequences obtained were used for domain analysis using MEME Suite [24], Pfam [25] and NCBI's Conserved Domains Database [26]. Multiple sequence alignment was performed in MEGA X program applying MUSCLE algorithm using default parameters [27]. The aligned DIR protein sequences were used for construction of phylogenetic trees using default parameters in MEGA X program applying neighbor joining algorithm [28].

### 2.2 Identification of potential miRNAs and their target DIR gene

**Fig. 1:**
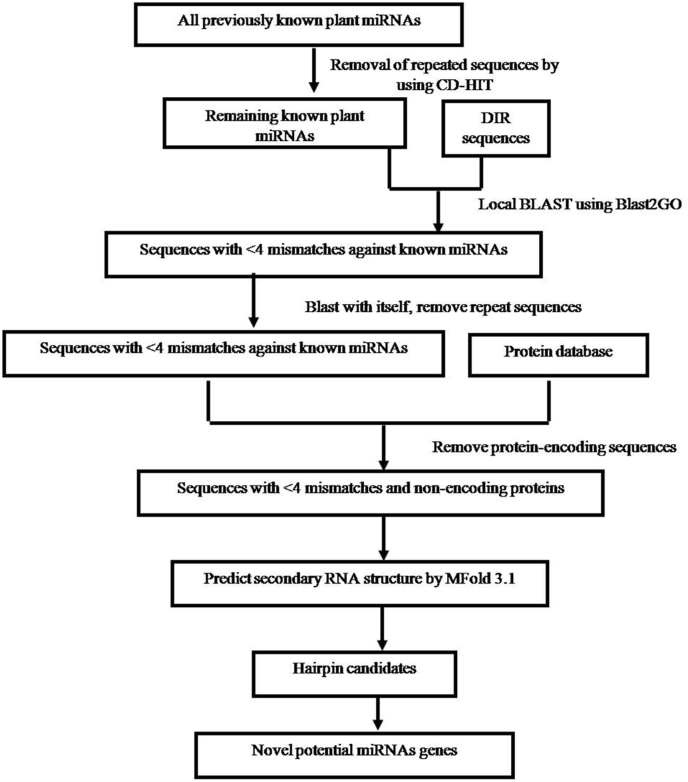
Workflow of the identification and characterization of potential miRNAs and their target DIR genes in *Oryza sativa* Indica.

Workflow of the identification and characterization of potential miRNAs, and target genes is depicted in Fig. 1. A total of 10898 mature miRNA sequences were retrieved from PMRD: Plant micro RNA Database (http://bioinformatics.cau.edu.cn/PMRD/) [29]. With identity value 90, CD-HIT-v4.5.4 was used to remove the redundancy in miRNA sequences [30]. In order to identify miRNA-targeted DIR genes of Indica rice, Local BLAST was performed using Blast2GO version 5.2 [31]. BLASTx analysis (E-value ≤ 1e^−10^) was performed in order to remove protein-coding sequences from precursor sequences.

### 2.3 Prediction of the secondary structure of pre-miRNAs

Prediction of the secondary structure was done by using the software MFOLD 3.1 [32] available at (http://www.bioinfo.rpi.edu/applications/mfold/rna/form1.cgi). The following criteria were used for screening the candidates of potential miRNAs: minimum length of the pre-miRNA to be 60 nt; pre-miRNA should be folded into appropriate stem-loop hairpin secondary structure; mature miRNA sequence should be placed in one arm of the hairpin structure; not >6 nt mismatches in miRNA/miRNA duplex; No loops or breaks between the miRNA/miRNA duplex; A+U content within 30–70%; Predicted secondary structure should have higher minimal folding free energy index (MFEI) and negative minimal folding free energy (MFE) values [33]. The MFE or ΔG (-kcal/mol) values generated from the MFOLD web server of the stem-loop structures were used for calculating the MFE index values using the following formula:

$$\text{MFEI}=\frac{\left(\text{MFE}/\text{length}\;\text{of}\;\text{precursor}\;\text{miRNA}\;\text{sequence}\right)\times 100}{\%\;\text{GC}\;\text{content}}$$

### 2.4 Plant growth and stress treatment

Seeds of *Oryza sativa* were surface sterilized with 70% ethanol for 1 min followed with 0.1% Mercuric chloride (HgCl_2_) for 5 min and then 0.2% Bavastin for 10 min. All seeds were placed in the dark for 2 days then allowed to germinate for 15 days under control condition maintained at 26 ± 2 °C with 16/8 h light/dark photoperiods cycle. Thereafter the seedlings were subjected to different abiotic stress for 48 h, which includes high temperature (48 °C), low temperature (4 °C), heavy metal (6 mM CdCl_2_·H_2_O), salinity (200 mM NaCl) and drought (15% polyethylene glycol).

### 2.5 Total RNA isolation and cDNA synthesis

Total RNA was isolated from various abiotic stress treated as well as untreated seedlings and were used as a template for the cDNA synthesis following the manufacturer's protocol (Fermentas, EU). Two sets of cDNA were synthesized and used for expression analysis. For DIR gene expression analysis, 2 μg total RNA was used for first-strand cDNA synthesis. However, for miRNA expression analysis, the total RNA was initially polyadenylated with Poly (A) Polymerase Tailing Kit following the manufacturer's protocol (Epicentre, USA; Cat. No. PAP5104H). The polyadenylated RNA was then used for the synthesis of cDNA using a reverse primer of specific miRNA. The cDNA synthesized was later used as a template for expression studies using quantitative real-time PCR (qRT-PCR).

### 2.6 Validation using quantitative real-time PCR

Gene-specific primers for all miRNAs and their DIR specific target gene were designed using miRPrimer [34] and OligoAnalyzer Tool 3.1 tool (Integrated DNA Technologies, Inc) respectively. The rice actin 1 gene was used as an internal control to normalize the gene expression level. The qRT-PCR was performed on an AriaMx Real-Time PCR System (Agilent Technologies). The total reaction volume was 10 μl which contained 5 μl of 2X KAPA SYBR FAST qPCR Master Mix Universal, 200 nM gene-specific primers and 0.5 μl of cDNA. The thermal cycle reaction conditions were 95 °C for 3 min, followed by 40 cycles of 95 °C at 10 s and then 57 °C for 30 s. A melting curve was generated at the end of 40 cycles for analyzing the specificity of each gene. The experiment was conducted with two independent biological replicates and three technical replicates for each sample. The relative gene expression of the individual gene was calculated via 2^^∧^−ΔΔCT^ [35].

### 2.7 Statistical analysis

All the experimental data are means of triplicates and represented as means ± standard deviation (SD). The significance was tested using SPSS (Statistical Package for the Social Sciences) software (version 21 for Windows; IBM Ltd., Japan) for calculating Students's t-test at significance level p ≤ 0.05.

## 3 Results

### 3.1 Identification and domain analysis of DIR family genes in rice

**Fig. 2:**
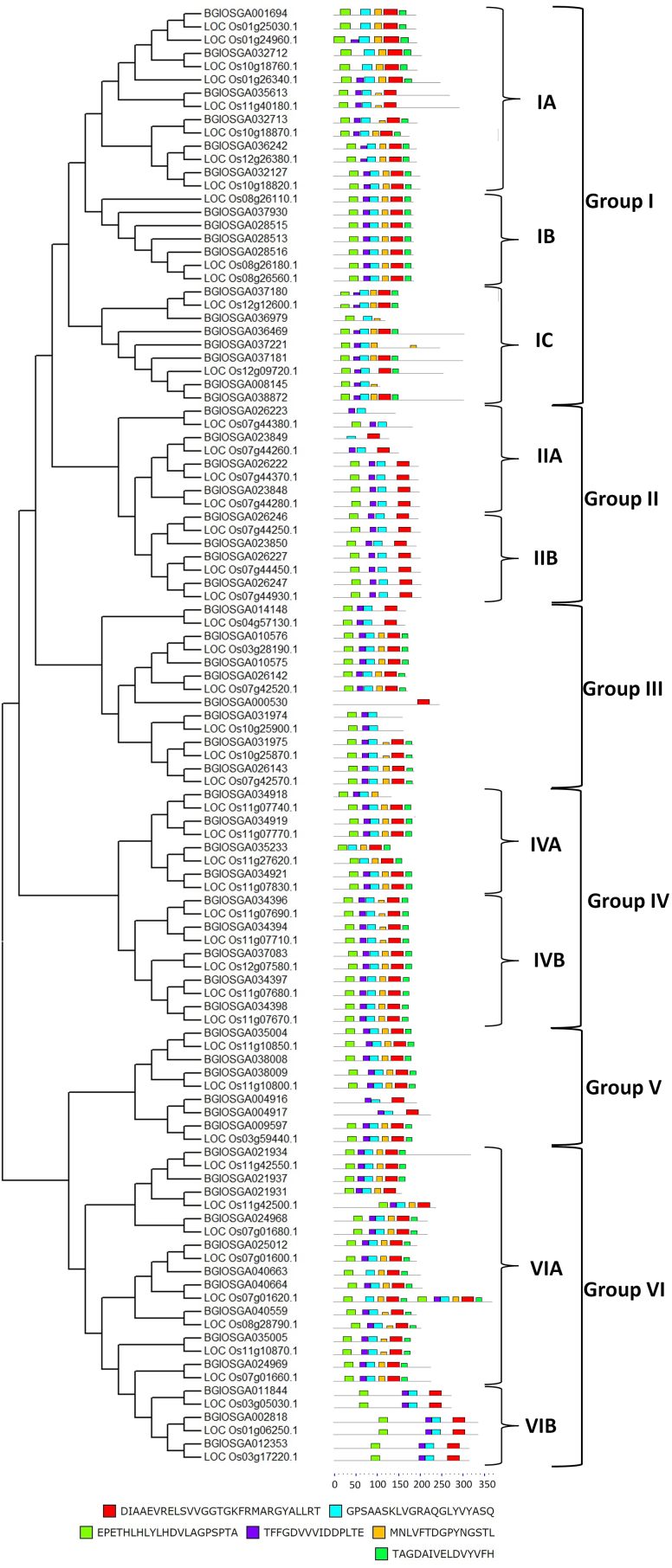
Phylogenetic tree analysis of the DIR protein sequences of Indica and Japonica rice.

In the present study, a total of 61 potential DIR family genes in rice were identified from Ensemble Plant database and thereafter their amino acid sequences were retrieved for further analysis (Table 1). The amino acid sequence of all theses 61 proteins showed the presence of the DIR domain (Accession No: PF03018) (Table 2). These 61 DIR proteins of Indica were aligned with 49 DIR proteins of Japonica rice as reported by Liao et al., 2016 [36]. The sequence homology was evident between the DIR proteins of Indica and Japonica rice as observed through the phylogenetic tree. The phylogenetic classification of DIR proteins revealed that the DIR proteins of Indica and Japonica rice can be divided into six major groups and in some cases into further 2–3 subgroups with characteristic motifs (Fig. 2).

**Table 1:** List of the DIR family proteins from Indica rice and their chromosome localization.

S.No.	Gene ID	Chromosome	Location	Gene (bp)		Protein (aa)	UniProtKB
1	BGIOSGA001694	1	15677624–15678515	510		169	A2WPQ9
2	BGIOSGA040664	1	7857–8486	630		209	*
3	BGIOSGA004916	1	41768710–41769297	588		195	A2WXK4
4	BGIOSGA000530	1	39337339–39338088	750		249	B8ABQ6
5	BGIOSGA040663	1	7012–7626	615		204	*
6	BGIOSGA004917	1	41771702–41772388	687		228	A2WXK5
7	BGIOSGA037930	1	12576–13550	567		188	*
8	BGIOSGA038009	1	15810–16391	582		193	*
9	BGIOSGA038008	1	1879–2430	552		183	A2ZCM2
10	BGIOSGA002818	1	3454855–3455877	1023		340	A2WKV1
11	BGIOSGA040559	1	15816–16403	588		195	*
12	BGIOSGA038872	1	573–2655	921		306	*
13	BGIOSGA008145	2	15968330–15968659	330		109	A2X4F8
14	BGIOSGA010575	3	18034326–18034862	537		178	A2XHQ3
15	BGIOSGA011844	3	2868738–2869565	828		275	A2XCF0
16	BGIOSGA010576	3	18014926–18015459	534		177	A2XHQ0
17	BGIOSGA009597	3	38189577–38190137	561		186	A2XN74
18	BGIOSGA012353	3	10452151–10453107	957		318	A2XF86
19	BGIOSGA014148	4	33138412–33139203	510		169	A2XYQ0
20	BGIOSGA021934	6	2661792–2664013	972		323	A2Y986
21	BGIOSGA021931	6	2690869–2691351	483		160	A2Y992
22	BGIOSGA021937	6	634860–2635369	510		169	A2Y980
23	BGIOSGA023849	7	24484280–24484748	393		130	A2YP28
24	BGIOSGA026143	7	23521390–23521959	570		189	A2YNQ7
25	BGIOSGA023850	7	24480425–24481012	588		195	A2YP27
26	BGIOSGA026142	7	23505182–23505700	519		172	A2YNQ5
27	BGIOSGA026223	7	24554378–24554818	441		146	B8B523
28	BGIOSGA025012	7	383064–383651	588		195	A2YHD4
29	BGIOSGA026227	7	24581749–24582366	618		205	A2YP43
30	BGIOSGA023848	7	24490027–24490635	609		202	A2YP29
31	BGIOSGA026246	7	24866340–24866936	597		198	A2YP80
32	BGIOSGA026222	7	24550944–24551546	603		200	A2YP37
33	BGIOSGA026247	7	24869603–24870223	621		206	A2YP81
34	BGIOSGA024968	7	410304–410966	663		220	A2YHD7
35	BGIOSGA024969	7	402155–402844	690		229	B8B6K1
36	BGIOSGA028515	8	17131391–17132365	567		188	A2YUB8
37	BGIOSGA028513	8	17024116–17025086	567		188	A2YUB1
38	BGIOSGA028516	8	17181235–17182205	567	188		A2YUC1
39	BGIOSGA032712	10	7828868–7829990	528	175		B8BG94
40	BGIOSGA032713	10	7848749–7849862	576	191		A2Z633
41	BGIOSGA031975	10	11789027–11789593	567	188		A2Z6Z9
42	BGIOSGA032127	10	7896440–7897612	615	204		A2Z638
43	BGIOSGA031974	10	11808382–11808867	486	161		A2Z700
44	BGIOSGA034918	11	3753012–3753422	411	136		A2ZC32
45	BGIOSGA035233	11	12317605–12318076	399	132		B8BKD9
46	BGIOSGA034919	11	3759307–3759861	555	184		A2ZC34
47	BGIOSGA034397	11	3720585–3721121	537	178		A2ZC28
48	BGIOSGA035004	11	5482044–5482595	552	183		A2ZCM2
49	BGIOSGA034396	11	3726825–3727358	534	177		A2ZC29
50	BGIOSGA035613	11	20564126–20567651	822	273		B8BLJ5
51	BGIOSGA034398	11	3717422–3717952	531	176		A2ZC27
52	BGIOSGA035005	11	5488104–5488649	546	181		A2ZCM3
53	BGIOSGA034394	11	3734798–3735334	537	178		A2ZC31
54	BGIOSGA034921	11	3806668–3807225	558	185		A2ZC38
55	BGIOSGA037180	12	6489050–6489637	465	154		A2ZJ62
56	BGIOSGA036979	12	2332122–2332618	369	122		A2ZI07
57	BGIOSGA036242	12	2551142–12552331	591	196		A2ZK73
58	BGIOSGA037083	12	4097865–4098419	555	184		A2ZIK4
59	BGIOSGA037181	12	6533842–6535164	915	304		A2ZJ67
60	BGIOSGA037221	12	7716317–7717733	753	250		B8BP28
61	BGIOSGA036469	12	5340966–5342358	924	307		A2ZIX4

*Currently not available.

**Table 2:** Domain analysis of DIR proteins of Indica rice.

S.No		Query		Hit type	PSSM-ID	From	To			E-Value	Bitscore		Accession	Short name
1		BGIOSGA001694		specific	335190	20	148			4.06E-36	122.24		pfam03018	Dirigent
2		BGIOSGA040664		specific	335190	39	182			3.48E-60	184.643		pfam03018	Dirigent
3		BGIOSGA032712		specific	335190	20	153			1.67E-30	107.988		pfam03018	Dirigent
4		BGIOSGA032713		specific	335190	22	169			2.04E-35	121.085		pfam03018	Dirigent
5		BGIOSGA034918		specific	335190	17	136			2.35E-61	184.643		pfam03018	Dirigent
6		BGIOSGA004916		specific	335190	45	182			1.59E-23	90.6541		pfam03018	Dirigent
7		BGIOSGA037180		specific	335190	24	148			3.94E-32	111.455		pfam03018	Dirigent
8		BGIOSGA035233		specific	335190	13	131			1.83E-59	179.635		pfam03018	Dirigent
9		BGIOSGA008145		superfamily	335190	26	97			1.97E-15	66.7717		cl03841	Dirigent superfamily
10		BGIOSGA000530		specific	335190	154	249			1.10E-06	46.7413		pfam03018	Dirigent
11		BGIOSGA040663		specific	335190	29	172			4.31E-48	153.827		pfam03018	Dirigent
12		BGIOSGA004917		superfamily	335190	62	217			1.33E-20	84.1057		cl03841	Dirigent superfamily
13		BGIOSGA023849		specific	335190	26	126			6.86E-25	91.8097		pfam03018	Dirigent
14		BGIOSGA036979		superfamily	335190	30	105			2.03E-13	62.1493		cl03841	Dirigent superfamily
15		BGIOSGA037930		specific	335190	40	181			5.79E-40	132.641		pfam03018	Dirigent
16		BGIOSGA028515		specific	335190	40	181			3.37E-37	125.707		pfam03018	Dirigent
17		BGIOSGA028513		specific	335190	40	181			6.13E-39	129.944		pfam03018	Dirigent
18		BGIOSGA028516		specific	335190	40	181			3.56E-38	128.018		pfam03018	Dirigent
19		BGIOSGA031975		specific	335190	36	184			8.58E-53	165.383		pfam03018	Dirigent
20		BGIOSGA026143		specific	335190	38	185			9.64E-47	149.975		pfam03018	Dirigent
21		BGIOSGA034919		specific	335190	41	183			5.13E-78	228.941		pfam03018	Dirigent
22		BGIOSGA038009		specific	335190	40	192			7.24E-53	165.768		pfam03018	Dirigent
23		BGIOSGA036242		specific	335190	35	177			7.38E-37	124.937		pfam03018	Dirigent
24		BGIOSGA032127		specific	335190	41	182			5.42E-42	138.419		pfam03018	Dirigent
25		BGIOSGA010575		specific	335190	27	175			1.19E-43	141.5		pfam03018	Dirigent
26		BGIOSGA023850		specific	335190	35		190		2.64E-43		141.5	pfam03018	Dirigent
27		BGIOSGA038008		specific	335190	34		182		3.29E-54		168.464	pfam03018	Dirigent
28		BGIOSGA034397		specific	335190	31		177		2.73E-68		204.288	pfam03018	Dirigent
29		BGIOSGA035004		specific	335190	34		182		3.29E-54		168.464	pfam03018	Dirigent
30		BGIOSGA034396		specific	335190	28		174		2.22E-74		219.311	pfam03018	Dirigent
31		BGIOSGA011844		specific	335190	143		251		1.02E-30		111.84	pfam03018	Dirigent
32		BGIOSGA026142		specific	335190	26		166		7.64E-48		151.901	pfam03018	Dirigent
33		BGIOSGA026223		specific	335190	1		105		1.08E-23		89.4985	pfam03018	Dirigent
34		BGIOSGA035613		specific	335190	19		160		5.87E-28		104.521	pfam03018	Dirigent
				superfamily	354810	233		271		2.61E-06		47.1472	cl21453	PKc_like superfamily
35		BGIOSGA037083		specific	335190	40		182		5.94E-75		221.237	pfam03018	Dirigent
36		BGIOSGA010576		specific	335190	30		174		2.81E-50		158.449	pfam03018	Dirigent
37		BGIOSGA034398		specific	335190	31		175		2.70E-65		196.584	pfam03018	Dirigent
38		BGIOSGA021934		specific	335190	34		168		5.19E-62		193.502	pfam03018	Dirigent
39		BGIOSGA021931		specific	335190	31		158		3.70E-55		170.005	pfam03018	Dirigent
40		BGIOSGA002818		specific	335190	211		306		5.86E-31		113.766	pfam03018	Dirigent
41		BGIOSGA009597		specific	335190	36		184		1.15E-69		207.755	pfam03018	Dirigent
42		BGIOSGA021937		specific	335190	33		167		3.08E-57		175.783	pfam03018	Dirigent
43		BGIOSGA035005		specific	335190	27		180		3.14E-52		163.457	pfam03018	Dirigent
44		BGIOSGA025012		specific	335190	36		176		1.92E-57		177.324	pfam03018	Dirigent
45		BGIOSGA026227		specific	335190	43		201		3.36E-39		131.1	pfam03018	Dirigent
46		BGIOSGA040559		specific	335190	36		181		4.85E-56		173.857	pfam03018	Dirigent
47		BGIOSGA012353		specific	335190	192		318		6.57E-35		123.781	pfam03018	Dirigent
48		BGIOSGA023848		specific	335190	45		198		1.11E-38		129.944	pfam03018	Dirigent
49		BGIOSGA026246		specific	335190	39		194		9.19E-42		137.648	pfam03018	Dirigent
50		BGIOSGA031974		specific	335190	35		118		1.63E-30		107.603	pfam03018	Dirigent
51		BGIOSGA026222		specific	335190	43		196		4.89E-43		140.73	pfam03018	Dirigent
52		BGIOSGA034394		specific	335190	31		175		7.18E-62		187.724	pfam03018	Dirigent
53		BGIOSGA026247		specific	335190	45		202		4.81E-42		138.419	pfam03018	Dirigent
54	BGIOSGA034921		specific		335190	41		184	3.28E-81		237.03		pfam03018	Dirigent
55	BGIOSGA024968		specific		335190	50		195	3.06E-69		208.14		pfam03018	Dirigent
56	BGIOSGA014148		specific		335190	26		164	4.19E-49		154.982		pfam03018	Dirigent
57	BGIOSGA037181		specific		187708	173		302	1.05E-47		155.802		cd09612	Jacalin
			specific		335190	25		149	5.84E-32		115.692		pfam03018	Dirigent
58	BGIOSGA037221		specific		187708	119		248	1.16E-40		136.157		cd09612	Jacalin
			superfamily		335190	26		106	8.10E-18		77.1721		cl03841	Dirigent superfamily
59	BGIOSGA038872		specific		187708	175		304	1.58E-38		132.305		cd09612	Jacalin
			specific		335190	26		149	3.77E-33		118.774		pfam03018	Dirigent
60	BGIOSGA036469		specific		187708	176		305	7.32E-41		138.468		cd09612	Jacalin
			specific		335190	25		149	4.27E-32		116.077		pfam03018	Dirigent
61	BGIOSGA024969		specific		335190	30		173	2.02E-67		203.903		pfam03018	Dirigent

### 3.2 Identification of miRNAs and their DIR specific target genes in rice

For the *in silico* prediction of potential rice miRNAs, a reference set of 10898 mature plant miRNAs was retrieved from the PMRD database (http://bioinformatics.cau.edu.cn/PMRD/). With identity value 90, CD-HIT-v4.5.4 was used to remove the redundancy in miRNA sequences. After removing redundant sequences, a set of 5025 miRNA sequences (reference set of miRNA sequences) were blasted (Local BLAST by using Blast2GO-v5.2) to the DIR genes assembly (Fig. 1).

Further, BLASTx analysis (E-value ≤1e^−10^) showed that out of 6 miRNA only 3 miRNA sequences were found to be non-coding indicating that they may play the role in *Oryza sativa* while the other 3 are coding for some protein. The putative miRNAs obtained had lengths of 21, 21 and 27 nucleotides for ath-miRf10317-akr, cre-miR910, and osa-miRf10761-akr, respectively (Table 3). Further, in order to identify miRNA-targeted DIR genes of rice, Local BLAST was performed using Blast2GO. The potential DIR specific targets of ath-miRf10317-akr, cre-mir910 and osa-miRf10761-akr were found to be BGIOSGA034397, BGIOSGA024969 and BGIOSGA036979, respectively (Table 3).

**Table 3:** Identified potential miRNA and its target DIR specific genes in Indica rice.

S. No.	miRNAs	DIR specific target genes
1	ath-miRf10317-akr	BGIOSGA034397
2	cre-miR910	BGIOSGA024969
3	osa-miRf10761-akr	BGIOSGA036979

### 3.3 Prediction of the secondary structure of potential miRNAs

**Fig. 3:**
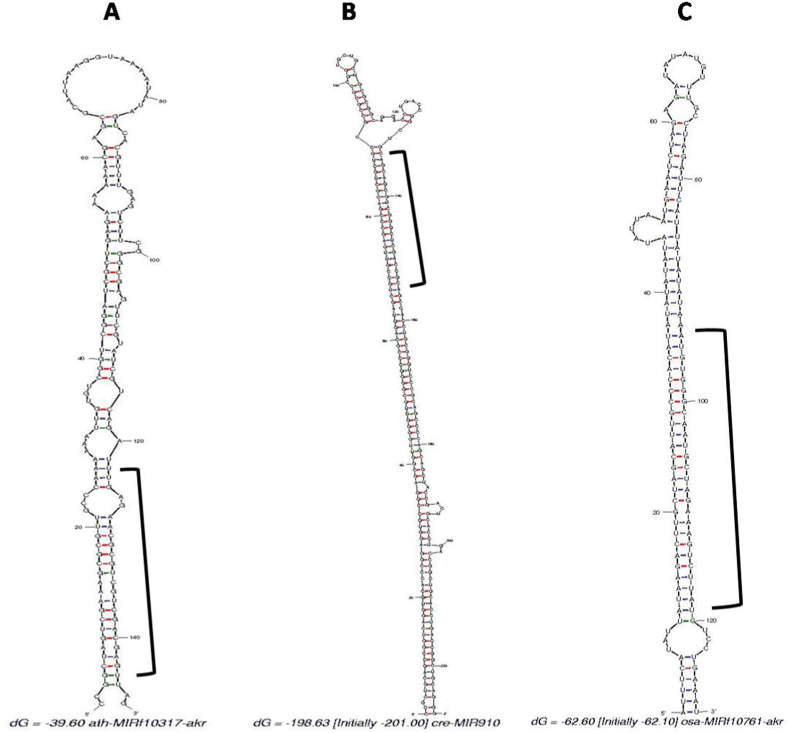
**A-C.** Mature and precursor sequences and the predicted stem-loop structures of identified miRNAs in *Oryza sativa* Indica Group- (A) ath-miRf10317-akr, (B) cre-miR910, and (C) osa-miRf10761-akr. The mature miRNAs are indicated with right square bracket.

The three non-coding miRNA sequences i.e., ath-mirf10317-akr, cre-mir910 and osa-mirf10761-akr, were further used for secondary structure analysis including hairpin stem-loop structure using MFOLD version 3.1. The negative MFE (-ΔG) of the miRNA precursors were also generated to study the stability of the hairpin stem-loop structure (Table 4). In comparison to the length of miRNAs, the length of putative precursor miRNAs of rice also varied significantly. It was observed as 227 nt, 129 nt and 147 nt for cre-miR910, osa-miRf10761-akr, and ath-miRf10317-akr, respectively (Fig. 3A–C). The stability of the secondary hairpin structure of pre-miRNA was determined by MFE (- ΔG). It was 211.20, 62.10 and 39.60 kcal/mol at 37 °C for cre-miR910, osa-miRf10761-akr and ath-miRf10317-akr, respectively. The distribution of G, C, A, and U nucleotides in the pre-miRNA were found to be different, where it ranged from 34.88 to 11.89% for A, 37.21–12.77% for U, 37.44–12.77% for G and 37.89–13.8% for C, respectively (Table 5). In the present investigation, AU content of miRNA cre-miR910 was found to be 25% which is below the 30–70% set range. Thus, miRNA cre-miR910 failed to qualify one of the eight criteria that we used to identify potential miRNA.

**Table 4:** Determination of minimal free folding energy (MFE) of the identified potential miRNA from Indica rice.

miRNAs	Mature miRNA sequence	Homologous miRNA	ST	Loc	LP	LM	NM	(G+C)%	MFE (ΔG)	AMFE	MFEI
ath-miRf10317-akr	GAGAACGCGUCGUCGACGAGU	hpo-mir-10102	+	3′	147	21	1	48.98	39.6	26.94	0.55
cre-miR910	AGCAGCGUCGGGCUCGACCGC	gga-mir-6557	+	3′	227	21	1	75.33	187.1	82.42	1.09
osa-miRf10761-akr	AAUGUGGGCAAUGCUAGAAAGUCUUAU	osa-MIR11336	+	3′	129	27	1	27.91	56.4	43.72	1.57

**Table 5:** The distribution of G, C, A, and U in the identified pre-miRNAs of Indica rice.

miRNAs	miRNA Family	A%	U%	G%	C%	A/U ratio	G/C ratio	(A+U)%
ath-miRf10317-akr	MiR10102	27.21	23.81	29.25	19.73	1.14	1.48	51.02
cre-miR910	miR910	11.89	12.77	37.44	37.89	0.93	0.99	24.67
osa-miRf10761-akr	miR11336	34.88	37.21	14.73	13.18	0.94	1.12	72.09

### 3.4 Seed germination and stress treatment

**Fig. 4:**
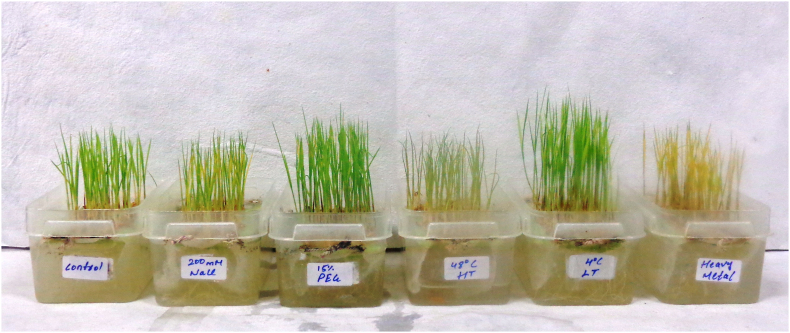
Effect of various abiotic stresses on 15 d old seedlings of Indica rice using hydroponic method.

Rice seedlings were germinated for 15 days followed by 48 h abiotic stress treatment which includes salinity, drought, heavy metal, high and low temperature. After 48 h, the seedlings which were exposed to stress treatment showed significant morphological changes (Fig. 4). Seedlings showed chlorosis of the shoot region, curling of leaves, necrosis in shoot and root tissue, and stunted growth of both shoot and root as compared to the untreated control seedlings.

### 3.5 Quantitative RT-PCR analysis

**Fig. 5:**
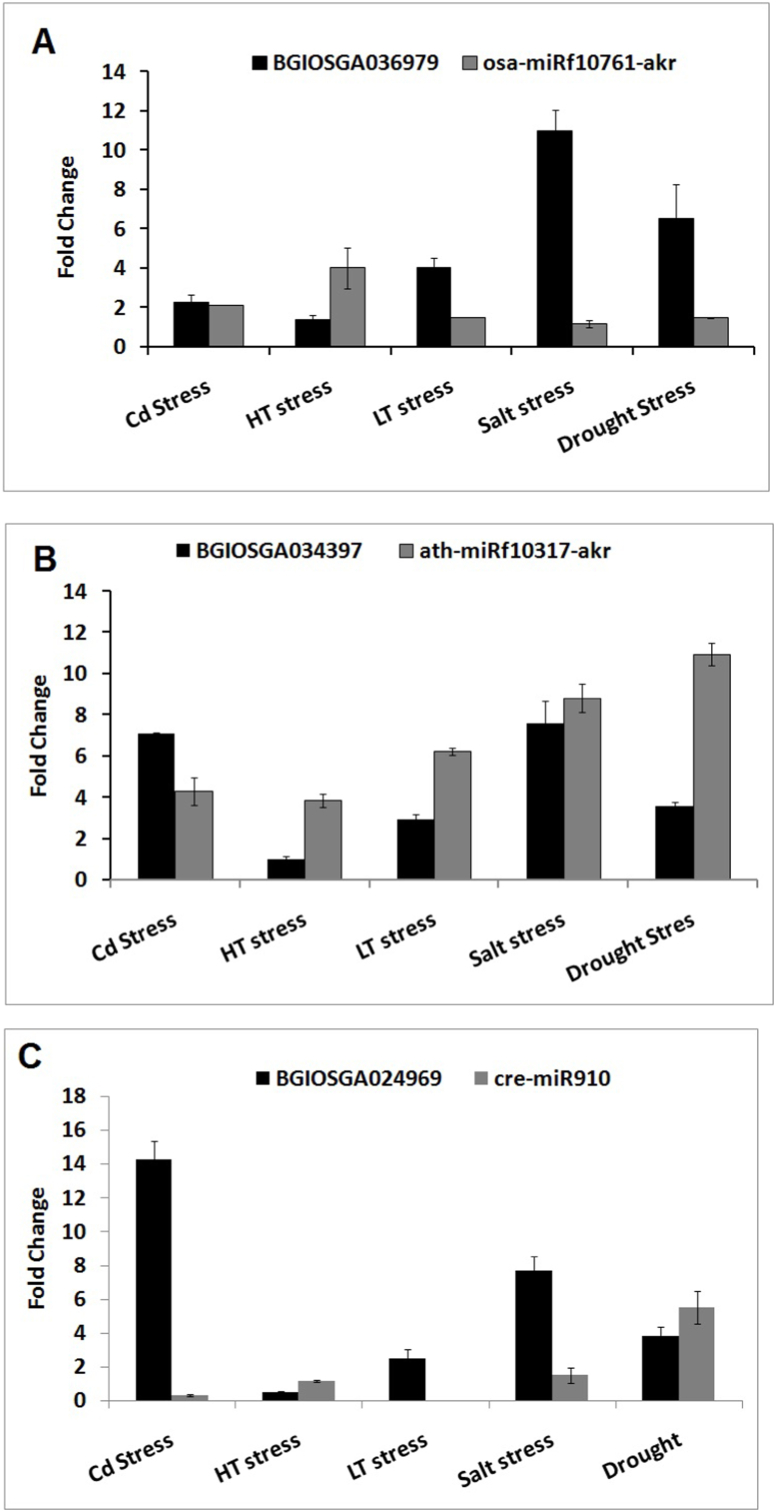
**A-C.** Expression analysis of miRNA (indicated in grey color) and their DIR specific target genes (indicated in black color) under different abiotic stress using quantitative Real Time PCR. (A) osa-miRf10761-akr miRNA and BGIOSGA036979 gene, (B) ath-miRf10317-akr miRNA and BGIOSGA034397 gene, and (C) cre-miR910 miRNA and BGIOSGA024969 gene.

The expression of miRNA and their DIR specific target genes under different abiotic stress conditions was analyzed using quantitative Real Time PCR. Expression of osa-miRf10761-akr miRNA and it targets DIR gene showed inverse expression pattern. Expression of osa-miRf10761-akr miRNA showed significant down-regulation (p ≤ 0.05) whereas its target DIR gene (BGIOSGA036979) showed significant up-regulation in response to drought, low temperature and salt stress. Under high temperature stress, expression of DIR gene (BGIOSGA036979) got significantly down-regulated whereas osa-miRf10761-akr miRNA showed significant up-regulation. However, the expression in response to heavy metal treatment was insignificant (Fig. 5A). Except heavy metal, expression of ath-miRf10317-akr miRNA showed significant up-regulation in response to drought, salinity, high and low temperature stress whereas it target DIR gene (BGIOSGA034397) showed significant down-regulation. Under heavy metal stress, the DIR gene and ath-miRf10317-akr miRNA showed inverse pattern (Fig. 5B). Expression of cre-miR910 miRNA showed significant down-regulation and its target DIR gene BGIOSGA024969 remained up-regulated in response to heavy metal, low temperature and salt treatment. Under high temperature and drought treatment, the expression of cre-miR910 miRNA was up-regulated while its target DIR gene was observed as down-regulated (Fig. 5C).

## 4 Discussion

Rice (*Oryza sativa* L.) is a model plant species, which is ranked second after maize in production globally. It is mostly cultivated in Asian countries like China, India, Indonesia, Bangladesh, Vietnam and Thailand. Rice is sensitive to various abiotic stresses which includes drought, salinity, heavy metals, high and low temperature. Rice in response to different abiotic stress undergoes several changes at the morphological, physiological, biochemical and molecular level [37,38].

Upon exposure to abiotic stress several genes/proteins gets up- or down regulated [[39], [40], [41]]. These proteins are believed to play vital role against biotic and abiotic stresses in plants. One such protein is the dirigent protein (DIRs) which is involved in lignifications. Lignin is mainly deposited in the vascular tissues during plant development and provides additional strength and protection to the cell wall. Liao et al., 2016 using the NCBI database reported genome-wide analysis of 49 DIR or DIR-likes genes in rice (*Oryza sativa* Japonica Group) [36]. However, in the present study, we have used Ensemble Plant database for the identification of DIR family genes in Indica rice. Using Pfam ID “PF03018” as keyword, we have identified a total of 61 DIR family genes in Indica rice. All the DIR proteins showed the presence of conserved dirigent domain (Accession No: PF03018) which is the characteristic feature of DIR family protein [2]. In pepper (*Capsicum annuum* L.), Pfam ID “PF03018” was used as keyword for the identification of dirigent gene family [42]. DIR proteins were also reported in numerous plant species, including lichens, ferns, gymnosperms, and angiosperms [[1], [2], [3]]. The phylogenetic classification along with the identified motifs, as shown in Fig. 2, distinctively identified six major groups of DIR proteins in both Indica and Japonica group of *Oryza sativa* named as DIR Group I to VI. All the groups showed the presence of 6 unique motifs except Group II and Group VIB which excluded the motif “MNLVFTDGPYNGSTL” marked in yellow. Some unique features could also be observed in both Indica as well as Japonica were there was loss of the characteristic motif(s) in the individual group members like BGIOSGA036979, BGIOSGA037221, BGIOSGA008145, BGIOSGA026223, LOC_Os07g44380.1, BGIOSGA023849, LOC_Os07g44260.1, BGIOSGA000530, BGIOSGA031974, LOC_Os10g25900.1, BGIOSGA004916, BGIOSGA004917, BGIOSGA021931, LOC_Os11g42500.1 and double occurance of all the 6 motifs in LOC_Os07g01620.1. The results in Table 2 where four dirigent superfamily members in Indica showed absence of the unique dirigent motif (PF03018) could also be corroborated with Fig. 2 where the dirigent motif “DIAAEVRELSVVGGTGKFRMARGYALLRT” marked in red was found absent in BGIOSGA036979, BGIOSGA037221, BGIOSGA008145 and BGIOSGA004917.

Earlier studies have shown that dirigent genes were expressed in different patterns in response to various abiotic stresses. In Japonica rice, expression of 13 OsDIR genes in response to dehydration, salinity and cold stresses were analyzed in rice seedling [36]. Expression analyses showed up or down regulation of OsDIR genes indicated that OsDIR genes are involved in the response process of abiotic stresses. In the present study, expression of three DIR genes i.e., BGIOSGA036979, BGIOSGA034397 and BGIOSGA024969 were analyzed in response to salinity, drought, high and low temperature and cadmium stresses in rice seedling. Differential expression showed low or high expression of DIR genes in response to various abiotic stresses. Expression of all the three DIR genes showed more than two-fold increase under cadmium, salinity and drought stress. Similar observation with two-fold down or up expression for selected sixteen OsDIR genes was also noted under at least one of the stress conditions studied in Japonica rice [36].

It is known that miRNAs regulate gene expression at the post-transcriptional level by translational repression or target degradation and gene silencing [[43], [44], [45]]. Although much progress has been made, the understanding of the molecular mechanism of abiotic stress tolerance remains insufficient [46]. Till now, many studies have revealed that rice miRNAs participate in various abiotic stress like drought [[47], [48], [49]], salinity [[50], [51], [52]], high temperature [53,54], low temperature [55], heavy metal [[56], [57], [58]], etc., indicating that miRNAs are involved in response to abiotic stresses.

We identified and characterized miRNA and their DIR specific targets in *Oryza sativa* var Indica using bioinformatics approaches. Using the Plant micro RNA Database, we obtained 3 miRNAs i.e., ath-mirf10317-akr, cre-mir910, osa-mirf10761-akr, and their respective DIR specific targets were BGIOSGA034397, BGIOSGA024969, BGIOSGA036979. In *Chlamydomonas reinhardtii*, cre-mir910 miRNA targets the NCR2 gene which gets up-regulated in stress to protect the cells from damage induced by stress [59]. In the present investigation, differential expression of ath-mirf10317-akr, cre-mir910, osa-mirf10761-akr, and their respective DIR specific targets genes in response to salinity, drought, cadmium, high and low temperature stresses in rice seedling were analyzed. In response to salinity, cadmium and low temperature stresses the expression of cre-mir910 and osa-mirf10761-akr miRNA were down-regulated compared to its respective DIR specific targets genes. Similar result was observed for the cre-mir910 miRNA where its expression was found down-regulated under multiple stress conditions in *C. reinhardtii* [59]. Role of miRNA ath-mirf10317-akr and osa-mirf10761-akr in various abiotic stresses is not reported till date. However, few reports showed the targets for miRNAs ath-mirf10317-akr codes for Synaptobrevin/vesicle-associated membrane protein (VAMP) (AT1G08820.2) [60] and several targets of miRNA osa-miRf10761-akr has been identified which includes inorganic H+ pyrophosphatase (LOC_Os06g08080.1), DEAD-box ATP-dependent RNA helicase (LOC_Os02g54020.1), PPR repeat containing protein (LOC_Os10g33700.2) and expressed protein (LOC_Os08g38620; LOC_Os09g15639) [61]. Thus, the identification and functional elucidation of miRNA targets are crucial to uncover the roles of miRNAs under abiotic and biotic stress [[62], [63], [64], [65]].

## 5 Conclusion

In the present study, we identified 3 miRNAs i.e., ath-mirf10317-akr, cre-mir910, osa-mirf10761-akr and their respective DIR specific targets genes i.e., BGIOSGA034397, BGIOSGA024969 and BGIOSGA036979, respectively. This is the first report in any organism showing the expression of ath-miRf10317-akr and osamiRf10761-akr miRNAs in response to various abiotic stresses. Several miRNA are known to regulate the expression of genes that are involved in abiotic stress tolerance. Expression of miRNA and it targets DIR gene showed inverse expression pattern in response to different abiotic stress treatment. We concluded that dirigent protein, which is involved in lignifications, plays an important role in abiotic stress response in plants.

## Funding

This investigation has been carried out under EMEQ grant awarded to RSP from the 10.13039/501100001843Science and Engineering Research Board (SERB), 10.13039/501100006143Department of Science and Technology, India (Grant No. EEQ/2016/000166) and Faculty Research Grant Scheme received from Guru Gobind Singh Indraprastha University, New Delhi, India (Grant No. GGSIPU/DRC/Ph.D/Adm./2017/514).

## CRediT author contribution statement

**Deepak Kumar Singh:** designed the research project and is the corresponding author, performed computational work, Formal analysis, and paper preparation. All the authors have read and agreed to publish the version of the manuscript. **Shourya Mehra:** designed the research project and is the corresponding author, performed computational work, Formal analysis, and paper preparation. All the authors have read and agreed to publish the version of the manuscript. **Sayan Chatterjee:** designed the research project and is the corresponding author, performed computational work, Formal analysis, and paper preparation. All the authors have read and agreed to publish the version of the manuscript. **Ram Singh Purty:** designed the research project and is the corresponding author, performed computational work, Formal analysis, and paper preparation. All the authors have read and agreed to publish the version of the manuscript.

## Declaration of competing interest

The authors declare that there are no conflicts of interest.
